# In Silico Analysis of Saroglitazar and Ferulic Acid Binding to Human Ketohexokinase: Implications for Metabolic Dysfunction-Associated Steatotic Liver Disease (MASLD)

**DOI:** 10.7759/cureus.79437

**Published:** 2025-02-22

**Authors:** Kalpana Tiwari, Brijesh Kumar, Anurag Tiwari, Puneet Dhamija, Gyan Vardhan, Amol Dehade, Vinay Kumar

**Affiliations:** 1 Pharmacology, Institute of Medical Science, Banaras Hindu University, Varanasi, IND; 2 Pharmacology and Therapeutics, Institute of Medical Science, Banaras Hindu University, Varanasi, IND; 3 Gastroenterology, Institute of Medical Science, Banaras Hindu University, Varanasi, IND; 4 Clinical Pharmacology, All India Institute of Medical Sciences, Rishikesh, IND; 5 Pharmacology, All India Institute of Medical Sciences, Rishikesh, IND; 6 School of Biotechnology, Center for Bioinformatics, Institute of Medical Science, Banaras Hindu University, Varanasi, IND

**Keywords:** chronic liver disease, fatty liver, ferulic acid, liver disease, mash, metabolic dysfunction-associated fatty liver disease (mafld), metabolic dysfunction-associated steatotic liver disease (masld), nafld treatment, saroglitazar

## Abstract

Background: Non-alcoholic fatty liver disease renamed as metabolic dysfunction-associated steatotic liver disease (MASLD) and a global health issue that causes excessive liver fat deposition without alcohol usage. Basic fatty liver to non-alcoholic steatohepatitis can lead to liver fibrosis, cirrhosis, and hepatocellular carcinoma. Role of research is vital due to the multifaceted, complex pathophysiology and the increasing incidence of a sedentary lifestyle. Computational network pharmacology, docking and dynamics studies of saroglitazar and ferulic acid with human ketohexokinase (KHK) were conducted to propose potential MASLD management.

Method: Utilized computational methodologies were utilized to examine binding interactions of saroglitazar (compound identifier (CID): 60151560) and ferulic acid (CID: 445858) with human ketohexokinase (KHK: P50053, Protein Data Bank (PDB) ID: 6W0W). Active site analysis was done by using the Conserved Domain Database (CDD) server (Collaborative Drug Discovery, Burlingame, California) and BIOVIA Discovery Studio 2019 (Dassault Systèmes, Vélizy-Villacoublay, France). The best PDB complex was used for molecular dynamics simulation and trajectory analysis on 100 ns, and functional associations were checked based on network analysis using the Search Tool for Interactions of Chemicals (STITCH) server (STITCH Consortium (EMBL), Heidelberg, Germany).

Results: Human ketohexokinase (KHK) protein (UniProt ID: P50053) was obtained. Additional KHK PDB Structure (6W0W) was retrieved for docking calculation. PubChem Database 2 Structure-Data File (SDF) files (National Center for Biotechnology Information (NCBI), U.S. National Library of Medicine, Bethesda, Maryland), ferulic acid (CID: 445858) and saroglitazar (CID: 60151560) were used as ligands. Active site residues were identified using the CDD server and BIOVIA Discovery Studio 2019. Further, identified active site residues, i.e., Arg^108^, Trp^225^, Glu^227^, Gly^229^, Ala^230^, Pro^246^, Pro^247^, Val^250^, Thr^253^, Gly^257^, Cys^282^, Gly^286^, and Cys^289 ^were used as potential active site for docking. D. E. Shaw Research Molecular Dynamics (DESMOND, Schrödinger, Inc., New York) was used for molecular dynamics simulation and trajectory analysis equilibrated after 40 ns in best-docked complex (saroglitazar (CID: 60151560) and KHK; binding energy: −21 kcal/mol).

Conclusion: The study shows that saroglitazar and ferulic acid are potent KHK inhibitors for metabolic diseases, including MASLD, suggesting multi-target treatments.

## Introduction

Non-alcoholic fatty liver disease is renamed as metabolic dysfunction-associated steatotic liver disease (MASLD). MASLD is a prevalent and growing global health concern characterized by excessive accumulation of fat in the liver without alcohol consumption [1,2]. This condition can progress from simple steatosis (fatty liver) to metabolic dysfunction-associated steatohepatitis (MASH). Non-alcoholic steatohepatitis (NASH), now replaced with the term metabolic dysfunction-associated steatohepatitis (MASH), can further lead to liver fibrosis, cirrhosis, and ultimately, hepatocellular carcinoma (HCC) [3,4]. The multifactorial nature and complex pathophysiology of MASLD, coupled with the increasing prevalence driven by sedentary lifestyles and unhealthy dietary habits such as calorie imbalance, where caloric intake exceeds energy expenditure, lead to obesity and metabolic syndrome, which are major risk factors for MASLD. High-calorie diets, particularly with those rich in fats and sugars, contribute to the development of obesity-related liver fat accumulation [5] and underscore the urgent need for comprehensive research efforts to better understand and develop effective therapeutic strategies [6]. MASLD has surged globally over the past 30 years, affecting one-third of the world's population, with Latin America and Middle East and North Africa (MENA) regions having the highest prevalence. However, it is a significant concern across the globe, with 25-35% of the population in other regions also affected by MASLD. India, now the most populous country globally, faces a significant burden of MASLD, with an estimated prevalence of 38.6% and notable geographic and rural-urban variations. Considering its large population and the high prevalence rate, India likely has one of the world's highest numbers of MASLD cases [1]. Despite this, effective treatments and awareness are lacking, with only 32 countries having national guidelines for MASLD and no comprehensive public health response in place. Recognizing MASLD as a significant non-communicable disease, the WHO is crucial for developing tailored policies to address its multifaceted impact on health, economics, and patient well-being, underscoring the urgent need for collective action [7,8]. However, the therapeutic options and strategy are very limited for MASLD.

Saroglitazar, a medication that simultaneously activates both peroxisome proliferator-activated receptor alpha (PPARα) and gamma (PPARγ), has been approved for the treatment of diabetic dyslipidemia (DD). However, this dual-acting agent holds promising therapeutic potential for addressing metabolic dysfunction-associated fatty liver disease (MASLD), a condition characterized by excessive fat accumulation in the liver unrelated to alcohol consumption [9,10]. Saroglitazar was approved in 2013 for marketing in India under treatment for atherogenic diabetic dyslipidemia [11]. Owing to its distinctive mechanism of action, saroglitazar demonstrated efficacy in managing metabolic dysfunction-associated steatotic liver disease (MASLD). Following successful clinical trials that validated its therapeutic benefits, the drug received regulatory approval in India as a treatment option specifically for MASLD [9,12-14].

Ferulic acid (FA), a phenolic acid abundant in plants, integrates with cell wall polysaccharides and proteins, forming its structure. Its diverse therapeutic benefits include hepatoprotective, antimicrobial, anti-inflammatory, and antioxidant properties at low concentrations and anti-tumor effects at higher concentrations or in the presence of metal ions, along with immune modulation [12,15,16]. The mechanism through which ferulic acid (FA) activates peroxisome proliferator-activated receptor alpha (PPARα) to prevent metabolic dysfunction-associated fatty liver disease (MASLD) involves the following key processes: Firstly, FA supplementation decreases the accumulation of triglycerides in the liver by promoting the breakdown of fatty acids through beta-oxidation and increasing the biosynthesis of ketone bodies, ultimately enhancing fatty acid expenditure [17,18]. Secondly, FA supplementation increases energy expenditure by facilitating the utilization of lipids as an energy substrate. These observations collectively suggest that FA may serve as a promising candidate for the prevention of MASLD as a synergistic effect with potential applications as a functional food or therapeutic agent [17]. Recent advancements in experimental and computational methods have facilitated successful drug repositioning (DR), consequently reducing the expenses, time, and risks associated with new drug discovery [19,20]. Computational approaches, including molecular docking, molecular dynamics (MD) simulation, and network pharmacology analysis, offer valuable insights into potential therapeutic agents' binding interactions and mechanisms of action. The study investigates the binding interactions of saroglitazar and ferulic acid with human ketohexokinase (KHK), an enzyme implicated in MASLD pathogenesis, aiming to propose potential therapeutic interventions for this debilitating condition. We collected information about compounds, compound-related targets, and MASLD-related genes from extensive databases and identified important targets and pathways using a protein-protein interaction network-based method.

## Materials and methods

Selecting and preparing saroglitazar and ferulic acid as ligands and human ketohexokinase (KHK) as the target protein. The 3D structures of these ligands were retrieved from the PubChem database, while the crystal structure of KHK was downloaded from the Protein Data Bank (PDB). Human ketohexokinase protein (UniProt ID: P50053) was obtained. Additional KHK PDB Structure (6W0W) was retrieved for docking calculation. PubChem Database 2 Structure-Data File (SDF) files (National Center for Biotechnology Information (NCBI), U.S. National Library of Medicine, Bethesda, Maryland), ferulic acid (compound identifier (CID): 445858) and saroglitazar (CID: 60151560), were used as ligands. Active site analysis was conducted using the Conserved Domain Database (CDD) server (Collaborative Drug Discovery, Burlingame, California) and BIOVIA Discovery Studio 2019 (Dassault Systèmes, Vélizy-Villacoublay, France). Molecular docking simulations were performed using BIOVIA Discovery Studio 2019, with various poses generated and evaluated based on binding affinity and interaction energy. Molecular dynamics simulations were performed using appropriate software, such as the D. E. Shaw Research Molecular Dynamics (DESMOND)-Maestro-2018.4 version (2023.2) (Schrödinger, Inc., New York), to observe the stability and behavior of the complexes.

Molecular dynamics simulation protocol

Simulation Method (Stepwise)

Simulation of the target protein/ligand complex was performed using the GROMACS simulation package (GROMACS Development Team, primarily KTH Royal Institute of Technology, Stockholm, Sweden) with the standard procedure (http://www.mdtutorials.com/gmx/). Simulation was conducted in four sequential stages:

Stage 1: Preprocessing, which includes protein and ligand topology generation. Ligand topology was created with our in-house customized toolkit that uses AMBERTOOLS and ACPYPE.

Stage 2: Energy minimization.

Stage 3: Equilibration of the simulation system was performed using the number of particles, volume, and temperature (NVT)/number of particles, pressure, and temperature (NPT) method, where volume and temperature were kept constant (NVT), followed by an additional equilibration step where pressure and temperature were kept constant (NPT).

Stage 4: A molecular dynamic simulation run was performed using a leap-frog integrator (MD).

MD simulation trajectories were processed using standard GROMACS results analysis packages. SiBioLead MD simulation module provides various widely used simulation trajectory analysis results for interpreting simulation run.

**Figure 1:**
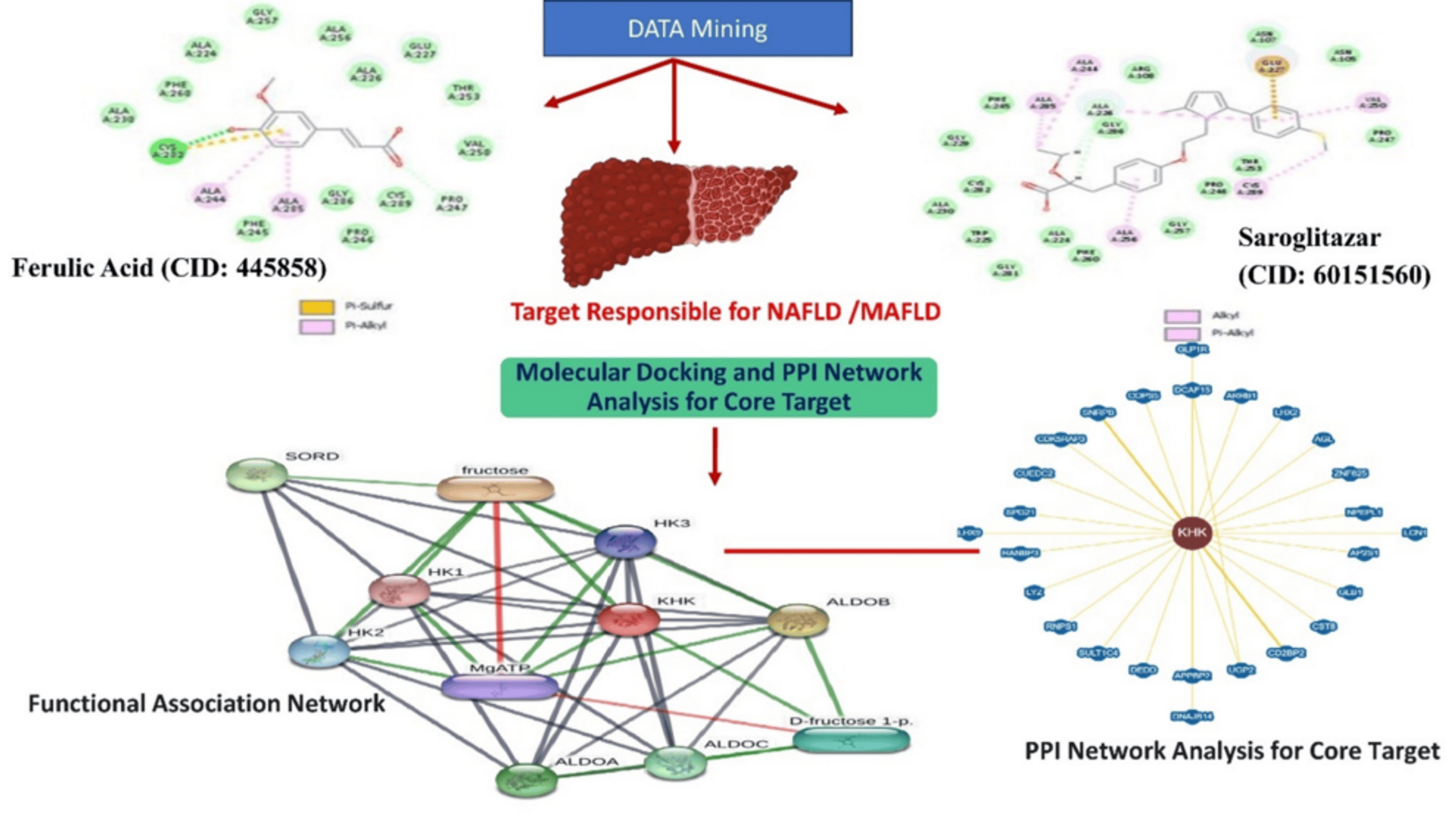
Graphical abstract. The image outlines a workflow combining computational and network-based methods to explore the interactions and efficacy of ferulic acid and saroglitazar in treating NAFLD/MASLD. Ketohexokinase (KHK) is a key target, indicating its potential for therapeutic intervention. PPI: protein-protein interaction, MASLD: metabolic dysfunction-associated steatotic liver disease, NAFLD: non-alcoholic fatty liver disease, ALDOA: aldolase A, ALDOB: aldolase B, ALDOC: aldolase C, SORD: sorbitol dehydrogenase, HK1: hexokinase 1, HK2: hexokinase 2, MgATP: magnesium adenosine triphosphate, KHK: ketohexokinase, CID: compound identifier.

DESMOND incorporates advanced algorithms for efficient simulation, including integration of Newton's equations of motion, force field calculations, and treatment of long-range interactions. The software's parallel computing capabilities enable high-performance simulations of large biomolecule systems. Trajectories obtained from MD simulations were analyzed to study the stability and conformational changes of the complexes. Functional associations of KHK in terms of biological processes and molecular functions were investigated using the Search Tool for Interactions of Chemicals (STITCH) server (STITCH Consortium (EMBL), Heidelberg, Germany). The latest available version is STITCH (5.0 2019). The results from STITCH were integrated with the molecular docking and MD simulation findings to provide a comprehensive understanding of the functional implications of the binding interactions (Figure 1).

## Results

**Figure 2:**
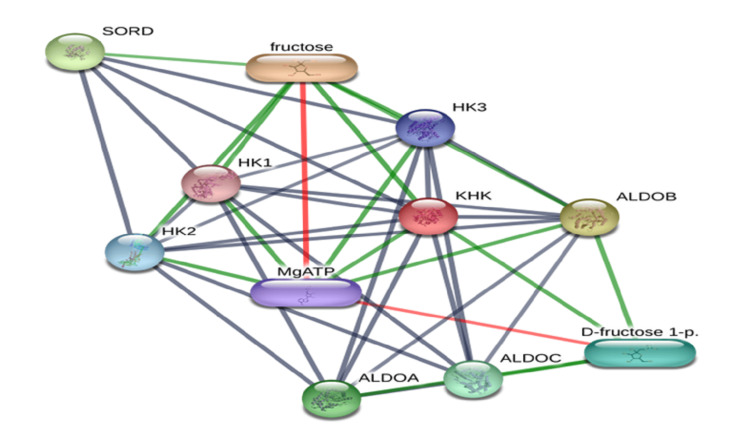
Functional association network potential therapeutic targets. HK1: hexokinase 1; HK2: hexokinase 2; MgATP: magnesium adenosine triphosphate; KHK: ketohexokinase; HK3: hexokinase 3; ALDOA: aldolase A; ALDOB: aldolase B; ALDOC: aldolase C; SORD: sorbitol dehydrogenase.

Human ketohexokinase (KHK) protein (UniProt ID: P50053) details were obtained. Based on details, KHK PDB Structure (6W0W) was retrieved for docking calculation. Ferulic acid (CID: 445858) and saroglitazar (CID: 60151560) SDF files were downloaded from the PubChem Database. Active site analysis was performed using the CDD server and BIOVIA Discovery Studio 2019. For docking analysis, several key residues in the active site have been identified on amino acid side chain A: Arg^108^, Trp^225^, Glu^227^, Gly^229^, Ala^230^, Pro^246^, Pro^247^, Val^250^, Thr^253^, Gly^257^, Cys^282^, Gly^286^, and Cys^289^. These residues likely play significant roles in ligand binding, with their interactions influencing the stability and strength of the complex. The DESMOND molecular dynamics simulation showed that the saroglitazar complex reached equilibrium after 40 ns, which suggests that the ligand stabilizes effectively within the active site. This time frame is significant because it implies that saroglitazar's binding is not only stable but also robust under physiological conditions. This equilibration process indicates that the ligand's binding affinity is strong and that its interactions with the active site residues are optimized. The network pharmacology approach consists of overlapped terms (genes or pathway terms)-based analysis, protein-protein interaction (PPI) network-based analysis, and PPI cluster identification. The PPI network analysis is the key and prerequisite to identifying core nodes with greater substantial contributions. Twelve proteins from PPI clusters were identified, and ten hub targets were identified: sorbitol dehydrogenase (SORD), hexokinase 1 (HK1), hexokinase 2 (HK2), magnesium adenosine triphosphate (MgATP), ketohexokinase (KHK), hexokinase 3 (HK3), aldolase A (ALDOA), aldolase B (ALDOB), aldolase C (ALDOC), and SORD and as potential therapeutic targets that were uploaded to the Search Tool for the Retrieval of Interacting Genes/Proteins (STRING)/STITCH protein database (Figure 2).

Protein and ligand preparation

The human ketohexokinase protein (UniProt ID: P50053) was used, with structural information obtained from the Protein Data Bank (PDB ID: 6W0W). Ferulic acid (CID: 445858) and saroglitazar (CID: 60151560) were obtained from the PubChem Database in SDF format. KHK's active site was investigated with the CDD server and BIOVIA Discovery Studio 2019, and critical residues were found in the binding.

Docking analysis

Docking studies identified the following residues as important inside the KHK active site: Arg^108^, Trp^225^, Glu^227^, Gly^229^, Ala^230^, Pro^246^, Pro^247^, Val^250^, Thr^253^, Gly^257^, Cys^282^, Gly^286^, and Cys^289^. These residues were discovered to serve important roles in the ligand-binding process, providing information about the molecular interactions between KHK and the ligands.

Molecular dynamics simulations

DESMOND molecular dynamics simulation and trajectory analysis were used to determine the stability of the ligand-protein complex (Table 1). The simulation revealed that the combination, including saroglitazar (CID: 60151560) and KHK, achieved equilibrium after 40 nanoseconds, demonstrating a stable interaction. This stability indicates that saroglitazar is a promising candidate for further research as a potential therapeutic agent targeting KHK regulation. The differences in binding energy between ligands are highly relevant to their therapeutic efficacy. A more negative binding energy typically signifies a stronger, more stable, and specific interaction with the target receptor, which is critical for ensuring sustained drug action, reducing side effects, and improving bioavailability. Drugs with stronger binding energies tend to be more potent, selective, and long-lasting, leading to better therapeutic outcomes with lower required doses. Thus, binding energy serves as a key predictor of a ligand's potential to succeed as a therapeutic agent. Table 1 compares the energy parameters for three ligands: ferulic acid (CID: 445858), saroglitazar (CID: 60151560), and RZA (CID: 154585749) that interact with a protein, including binding energy, ligand energy, protein energy, complex energy, and entropic energy. RZA (154585749) has a good binding affinity and acts as a control for this study. 445858 and 60151560, both candidates, show binding energies of −24.8013 and −21.6552, respectively. The most stable ligand energy (−58.1411 kcal/mol) implies that it forms the most stable protein-ligand complex (−13711.3536 kcal/mol) while having relatively high entropic energy (19.8602 kcal/mol). Ligand 445858 follows closely in binding affinity and complex stability, while ligand 60151560 has the lowest affinity and stability but the highest entropic energy (20.5350 kcal/mol).

**Table 1:** Molecular docking and simulation. Compares the energy parameters for three ligands (ferulic acid (CID: 445858), saroglitazar (CID: 60151560), and RZA (CID 154585749)), and that interact with a protein, including binding energy, ligand energy, protein energy, complex energy, and entropic energy. RZA (154585749) also has a good binding affinity and acts as a control for this study. 445858 and 60151560, both candidates show binding energy −24.8013 and −21.6552, respectively.

Ligand name	Binding energy (kcal/mol)	Ligand energy (kcal/mol)	Protein energy (kcal/mol)	Complex energy (kcal/mol)	Entropic energy (kcal/mol)
445858	−24.8013	−30.5362	−13627.71	−13683.0475	18.3324
15458749	−25.5025	−58.1411	−13627.71	−13711.3536	19.8602
60151560	−21.6552	−10.3622	−13627.71	−13659.7274	20.535

LibDock Score is a computational value used to assess how well a ligand binds to a protein in Schrödinger's Glide docking software. It is calculated by considering various energy factors, including van der Waals interactions, electrostatics, hydrogen bonds, desolvation effects, and shape complementarity. The score reflects the binding strength, with more negative values indicating better binding. This helps rank ligands, guiding researchers to identify the most promising candidates for further development. Table 2 compares the compounds with the higher LibDock Score compound 60151560, with the highest score, which is the most promising KHK-targeting compound for metabolic illnesses like metabolic-associated fatty liver disease (MAFLD). Compound 15458749 acts as a control, whereas compound 445858, despite its lower score, may be useful in combination therapies or structural alterations to boost binding affinity. Tables 2, 3 revealed significant insights through both CDOCKER (Discovery Studio® 3.1, Accelrys, Inc., San Diego, California) Potential Energy calculations and LibDock Score assessments. In the CDOCKER analysis (Table 3), compound 60151560 demonstrated the highest potential energy at 32.3494 kcal/mol, indicating superior binding stability with the KHK protein. This was followed by compound 445858 with 26.2539 kcal/mol, while 15458749 showed the lowest energy at 4.94779 kcal/mol. These energy values suggest varying degrees of protein-ligand interaction stability, with compound 60151560 forming the most stable complex.

**Table 2:** Compounds with higher LibDock Scores. The table compares the compounds with the higher LibDock Score compound 60151560, with the highest score, which is the most promising KHK-targeting compound for metabolic illnesses like MASLD. Compound 15458749 acts as a control, whereas compound 445858, despite its lower score, may be useful in combination therapies or structural alterations to boost binding affinity. The LibDock Score analysis further reinforced these findings, with compound 60151560 achieving the highest score of 162.98, significantly outperforming both 15458749 (124.928) and compound 445858 (85.282). KHK: ketohexokinase, MASLD: metabolic dysfunction-associated steatotic liver disease.

Number	Compounds	LibDock Score
1	60151560	162.98
2	15458749	124.928
3	445858	85.282

**Table 3:** CDOCKER potential energy of compounds with KHK. KHK: ketohexokinase.

Compounds	CDOCKER potential energy (kcal/mol)
60151560	32.3494
15458749	4.94779
445858	26.2539

Network pharmacology approach

A network pharmacology technique was used to identify potential treatment targets (Figure 2). This method involved overlapping gene or pathway term analysis, protein-protein interaction (PPI) network analysis, and cluster identification. The PPI network is critical for finding core nodes that contribute significantly to the overall network dynamics. Ten PPI clusters were discovered, with ten hub targets highlighted. The targets are HK1, HK2, MgATP, KHK, HK3, ALDOA, ALDOB, ALDOC, and SORD. These targets represent possible therapeutic intervention points and have been added to the STRING protein database for future investigation and validation.

## Discussion

A sedentary lifestyle and food habits are the main leading causes of MASLD. Simple ketonic sugars like fructose can be found in a variety of foods, including honey, root vegetables, and fruits. It is frequently used in food products as a sweetener [7]. It is quickly absorbed in the small intestine and subsequently transferred to the liver cells by the SLC2A9, Glut2, or Glut5 transporters. After fructokinase converts fructose to fructose-1-phosphate while consuming adenosine triphosphate (ATP), intracellular phosphorylate is depleted, adenosine monophosphate (AMP) deaminase is activated, and uric acid is produced. 

Glycerol-3-phosphate and acyl-coenzyme A, the metabolites of fructose-1-phosphate, are converted to triglycerides and long-chain fatty acids, respectively [21]. In contrast to the glucose metabolism route, fructokinase is not subject to negative feedback. Human metabolic disorders such as obesity, insulin resistance, hyperlipidemia, metabolic dysfunction-associated fatty liver disease (MASLD), and non-alcoholic steatohepatitis have all been linked to long-term excessive fructose consumption (MASH) [22,23]. Ketohexokinase (KHK) is an enzyme involved in fructose metabolism, specifically in the phosphorylation of fructose to fructose-1-phosphate. There are two types of KHK: KHK-A and KHK-C, encoded by different genes. Their role, mechanism, and importance in metabolic dysfunction-associated fatty liver disease (MASLD) are significant [24]. The metabolism of fructose by KHK in the liver leads to the production of intermediates that contribute to lipid accumulation, insulin resistance, and inflammation, all of which are key features of MASLD. In MASLD, increased fructose consumption can lead to elevated levels of fructose-1-phosphate, promoting de novo lipogenesis and triglyceride accumulation in hepatocytes, leading to fatty liver condition and disease sequelae [21]. This study intended to explore the interaction of human ketohexokinase (KHK) with diverse ligands, concentrating on docking, molecular dynamics simulations, and network pharmacology analysis to find novel therapeutic targets.

Targeting KHK activity and downregulation that inhibits the production of the intermediate product of fructose metabolism is proposed as a potential therapeutic strategy for MASLD. Understanding KHK's role in fructose metabolism and its implications for MASLD underscores the importance of dietary interventions and lifestyle modifications in the prevention and management of metabolic diseases. In this study, the molecular docking of the residues (Table 4)-Arg^108^, Trp^225^, Glu^227^, Gly^229^, Ala^230^, Pro^246^, Pro^247^, Val^250^, Thr^253^, Gly^257^, Cys^282^, Gly^286^, and Cys^289^-were identified as potential active site residues for docking calculation.

**Table 4:** Reported active site residues and common for saroglitazar. The reported active site residues for saroglitazar show a high level of consistency, with key residues like Cys:A^289^, Thr:A^253^, Ala:A^224^, Gly:A^286^, Phe:A^245^, and Ala:A^244^ frequently present. These residues are crucial for the drug's binding and functionality, highlighting their importance in the drug's mechanism of action.

Reported active site residues	Saroglitazar	Common
Val:A^250^, Cys:A^289^, Thr:A^253^, Arg:A^108^, Phe:A^260^, Ala:A^285^, Ala:A^224^, Ala:A^226^, Gly:A^257^, Trp:A^225^, Ala:A^230^, Gly:A^229^, Cys:A^282^, Gly:A^286^, Phe:A^245^, Glu:A^227^, Pro:A^246^, Pro:A^247^, Ala:A^244^, Ala:A^256^.	Glu:A^227^, Asn:A^107^, Arg:A^108^, Ala:A^244^, Ala:A^285^, Ala;A^226^, Gly:A^286^, Phe:A^245^, Gly:A^229^, Cys:A^282^, Ala:A^230^, Trp:A^225^, Ala:A^224^ , Gly:A^281^, Phe:A^260^, Cys:A^289^, Thr:A^253^, Pro:A^247^, Val:A^250^, Asn:A^250^, Asn:A^105^.	Cys:A^289^, Thr:A^253^, Glu:A^227^, Trp:A^225^, Ala:A^224^, Ala:A^244^, Pro:A^247^, Phe:A^245^, Gly:A^286^, Cys:A^282^, Gly:A^229^, Phe:A^260^, Arg:A^108^, Val:A^250^.

Tables 4, 5 highlight the active site residues involved in the interaction with saroglitazar and ferulic acid, respectively, identifying both unique and shared binding residues. 

**Table 5:** Reported active site residues and common for ferulic acid. Ferulic acid shares key active site residues, including Ala:A^230^, Phe:A^260^, Gly:A^257^, Glu:A^227^, and others, indicating their importance in binding and interaction for both compounds.

Reported active site residue (RZA)	Ferulic acid	Common
Val:A^250^, Cys:A^289^, Thr:A^253^, Arg:A^108^, Phe:A:^260^, Ala:A^285^, Ala:A^224^, Ala:A^226^, Gly:A^257^, Trp:A^225^, Ala:A^230^, Gly:A^229^, Cys:A^282^, Gly:A^286^, Phe:A^245^, Glu:A^227^, Pro:A^246^, Pro:A^247^, Ala:A^244^, Ala:A^256^.	Ala:A^230^, Phe:A^260^, Ala:A^224^, Gly:A^257^, Ala:A^256^, Glu:A^227^, Ala:A^226^, Thr:A^253^, Val:A^250^, Pro:A^247^, Cys:A^289^, Gly:A^286^, Ala:A^285^, Ala:A^244^, Aya:A^282^.	Ala:A^230^, Phe:A^260^, Gly:A^257^, Glu:A^227^, Ala:A^226^, Thr:A^253^, Val:A^250^, Aya:A^282^, Ala:A^244^, Ala:A^285^.

In Table 4, saroglitazar interacts with key residues such as Cys:A^289^, Thr:A^253^, Glu:A^227^, Trp:A^225^, Ala:A^224^, Ala:A^244^, Pro:A247, Phe:A^245^, Gly:A^286^, Cys:A^282^, Gly:A^229^, Phe:A^260^, Arg:A^108^, and Val:A^250^, which are also part of the reported active site, indicate critical interaction points that stabilize the ligand within the binding pocket. Similarly, Table 5 shows that ferulic acid interacts with residues like Ala:A^230^, Phe:A^260^, and Gly:A^257^, Glu:A^227^, Ala:A^226^, Thr:A^253^, Val:A^250^, Aya:A^282^, and Ala:A^285^, which are also common with the active site, suggesting important binding interactions that contribute to its stability and affinity.

**Figure 3:**
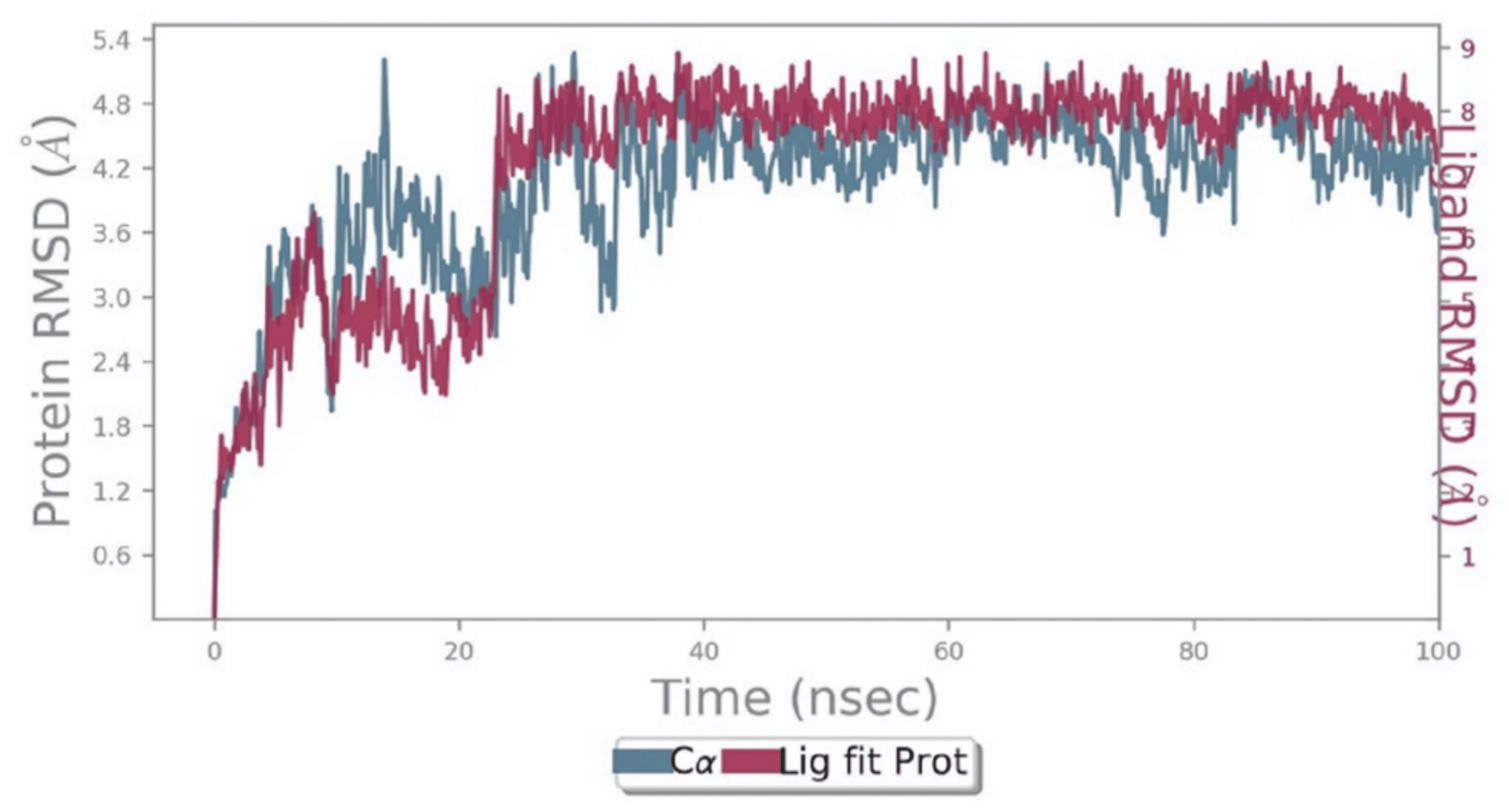
The root mean square deviations (RMSD) of backbone atoms relative to the starting complexes during 40 ns MD. The root mean square deviations (RMSD) plot for the backbone atoms relative to the starting complexes during a 40 ns molecular dynamics (MD) simulation provides valuable insights into the stability and dynamics of the system involving saroglitazar and ferulic acid. Initially, there is a rise in RMSD for both the protein backbone (Cα) and the ligand (Lig fit Prot) within the first 10 ns, indicating a phase of structural adjustment as the system equilibrates.

**Figure 4:**
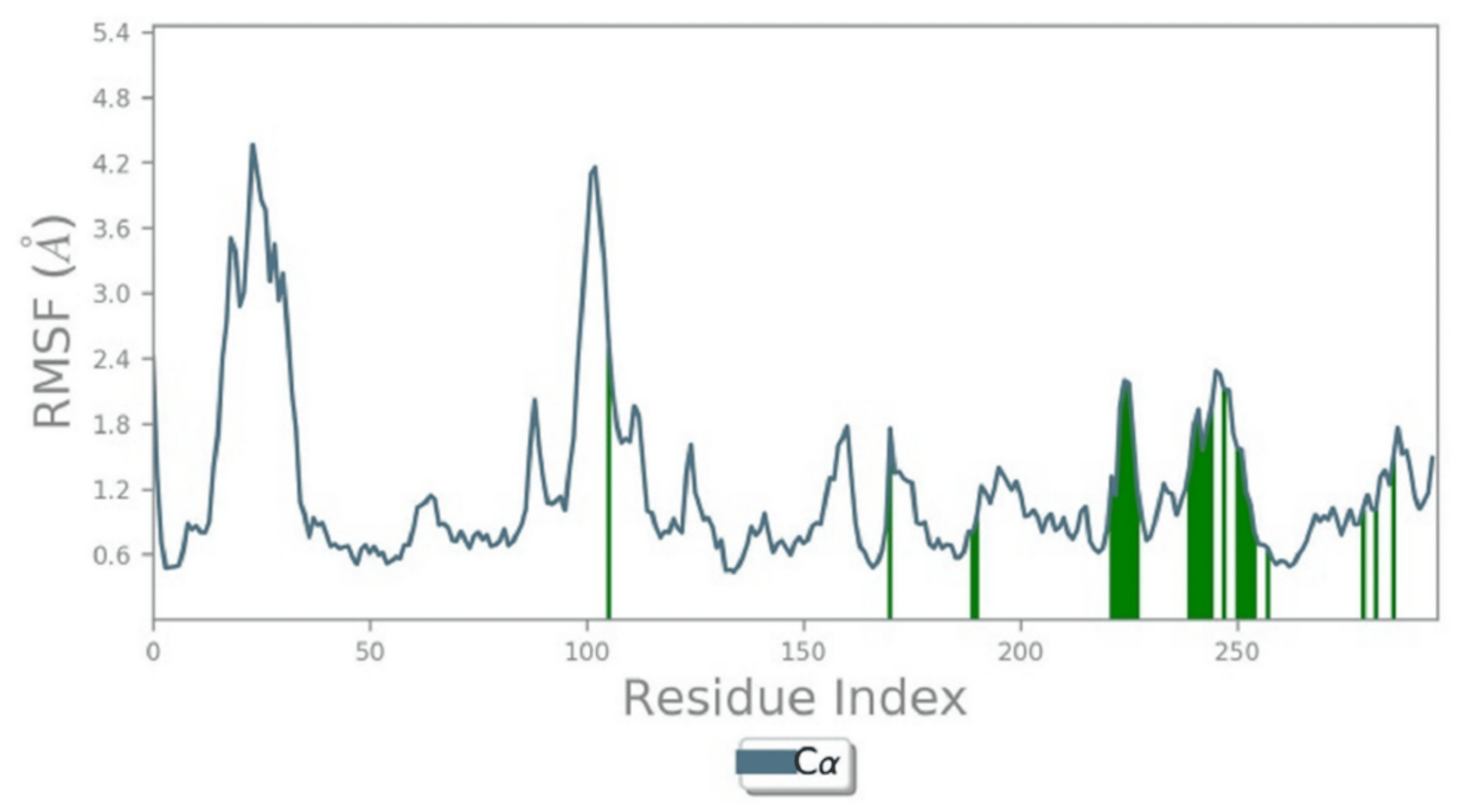
Root mean square fluctuation (RMSF) measures the average deviation of 40 ns MD. Root mean square fluctuation (RMSF) measures the average deviation of atomic positions from their mean positions over time in molecular dynamic simulations. It shows protein structural flexibility and dynamics. High-RMSF regions are flexible, while low-RMSF parts are rigid. The figure shows an RMSF plot of the complex with interacted active residues heightened in green. MD: molecular dynamics.

Both compounds share several residues, including Thr:A^253^, Val:A^250^, and Phe:A^260^, indicating similarities in their binding mechanisms. However, each compound also engages with unique residues, reflecting differences in their binding modes and the specific conformational changes they may induce in the protein. These findings provide valuable insights into the molecular interactions of saroglitazar and ferulic acid, enhancing the understanding of their potential as therapeutic agents targeting the same protein. Figures 3, 4 provide detailed insights into the molecular dynamics (MD) simulation and structural flexibility of the protein complex. Figure 3 presents the root mean square deviation (RMSD) of backbone atoms during the 40 ns MD simulation, which measures the stability of the complex relative to the initial structure.

Figure 4 displays the root mean square fluctuation (RMSF) of residues, indicating the average deviation of atomic positions over time. Higher RMSF values represent flexible regions of the protein, while lower values indicate more rigid areas. Figure 3 further elaborates on the RMSF plot, highlighting the fluctuating residues in green, which are involved in active interactions. This structural analysis helps identify regions of the protein that are critical for maintaining stability and interactions, providing a better understanding of its dynamic behavior in the context of molecular binding and potential therapeutic applications. 

Network pharmacology is an approach to intricate connections among medications, biological components, and diseases. The interactions between saroglitazar and ferulic acid with different enzymes and metabolites involved in metabolic dysfunction-associated fatty liver disease (MASLD), such as SORD, HK1, HK2, MgATP, KHK, HK3, ALDOB, ALDOC, and fructose-1-phosphate (F1P) (Figure 2). A thorough simulation examined the interactions between ketohexokinase (KHK), ferulic acid, and saroglitazar. The simulation found equilibrium for KHK with ferulic acid at a 100 ns time score and equilibrated at 40 ns. These equilibration timeframes show the time needed for the system to stabilize and appropriately analyze molecular interactions. The study examines the interaction between saroglitazar and ferulic acid with MASLD enzymes and metabolites, specifically SORD. SORD converts sorbitol into fructose, leading to liver lipogenesis and oxidative stress. Saroglitazar regulates hexokinase activity, which may boost glucose metabolism by reducing lipogenesis substrate availability. Ferulic acid may improve insulin sensitivity and lower hexokinase workload. Saroglitazar regulates MgATP levels to promote mitochondrial function and slow MASLD progression. MASLD requires adequate ATP for enzymatic activity, including glucose and lipid metabolism.

Aldolases, such as ALDOA, ALDOB, and ALDOC, break down fructose-1, 6-bisphosphate during glycolysis, causing MASLD [25,26]. The comparison of compounds based on their LibDock Scores reveals that compound 60151560, with a score of 162.98, is the most promising KHK-targeting compound for metabolic diseases such as MASLD. Its high score suggests a strong binding affinity, making it a prime candidate for further research and development. Compound RZA, with a score of 124.928, serves as a control in this comparison. Although it doesn't have the highest score, it still demonstrates significant binding affinity and can be used as a reference point for evaluating other compounds. Compound 445858, with a score of 85.282, has the lowest score among the three (Tables 2, 3). Despite this, it may still hold potential in combination therapies or through structural modifications to enhance its binding affinity, indicating that it shouldn't be disregarded solely based on its lower score. Saroglitazar may lower F1P and MASLD by enhancing insulin sensitivity and lowering lipogenesis. Antioxidant ferulic acid may prevent F1P-induced oxidative damage and inflammation. Fructose metabolism requires F1P, which increases fat synthesis and causes fatty liver disease, oxidative stress, and inflammation. Ketohexokinase (KHK)'s physiological activities support its use as a MASLD treatment target. KHK is essential to fructose metabolism, and its overactivity has been linked to metabolic diseases, including MASLD [27].

MASLD is chronic and complex; thus, targeting KHK may be a unique way to slow its development. Results from in silico and MD studies. We found substantial binding interactions between KHK and two possible medicinal drugs, saroglitazar and ferulic acid, in our in silico virtual screening and molecular dynamics simulation. These chemicals have substantial binding affinities and prolonged interactions with KHK's active site, suggesting they may impede KHK function. Typical active site residues, the main KHK-interacting active site residues for saroglitazar were: Cys:A^289^, Thr:A^253^, Glu:A^227^, Trp:A^225^, Ala:A^224^, Ala:A^244^, Pro:A^247^, Phe:A^245^, Gly:A^286^, Cys:A^282^, Gly:A^229^, Phe:A^260^, Arg:A^108^, Val:A^250^. Ferulic acid's main interacting residues were Ala:A^230^, Phe:A^260^, Gly:A^257^, Glu:A^227^, Ala:A^226^, Thr:A^253^, Val:A^250^, Aya:A^282^, Ala:A^244^, and Ala:A^285^, which were frequent residues in saroglitazar and ferulic acid, showing these amino acids are essential for binding and inhibition (Tables 4, 5). This overlap suggests that these residues are essential for KHK inhibition, making them promising therapeutic targets [28]. The combined targeting of key residues by saroglitazar and ferulic acid suggests a synergistic treatment strategy.

Ferulic acid is antioxidant and anti-inflammatory, whereas saroglitazar, a dual PPAR agonist, improves lipid metabolism and reduces hepatic steatosis. These drugs may diminish fructose-induced lipogenesis and oxidative stress by blocking KHK, two key MASLD processes. Saroglitazar has been shown to treat hypertriglyceridemia and improve insulin sensitivity, which are critical to MASLD therapy. Ferulic acid reduces oxidative stress and inflammation, supporting its MASLD treatment potential. These medications targeting KHK's common active site residues suggest a possible multi-target treatment. Saroglitazar and ferulic acid interact with KHK, notably at the shared active site residues, justifying its usage in MASLD treatment. A comprehensive treatment strategy can alter metabolic pathways, decrease oxidative stress and limit lipogenesis. In-vivo and clinical research are needed to confirm these findings and develop viable treatment strategies. In silico studies are limited by simplified models, data quality, and challenges in predicting absorption, distribution, metabolism, excretion, and toxicity (ADMET) properties, off-target effects, and biological complexities. Identifying and validating novel targets using in silico methods alone can be speculative, requiring experimental follow-up to confirm findings.

## Conclusions

This study shows that saroglitazar and ferulic acid are strong inhibitors of human ketohexokinase (KHK), which is a crucial enzyme in fructose metabolism. Molecular docking, molecular dynamics simulations, and functional association analysis revealed that both drugs have substantial binding affinities and persistent interactions with KHK's active site. These findings indicate that saroglitazar and ferulic acid may effectively suppress KHK activity, thereby providing therapeutic benefits for metabolic illnesses such as metabolic dysfunction-associated steatotic liver disease (MASLD). The multi-target therapeutic potential of these inhibitors shows their promising role for the treatment of complicated metabolic illnesses, requiring additional exploration.
